# Supplementary material for: An automated single-molecule FRET platform for high-content, multiwell plate screening of biomolecular conformations and dynamics
Source: Nat Commun. 2023 Oct 16;14:6511. doi: 10.1038/s41467-023-42232-3 (PMC10579363; doi:10.1038/s41467-023-42232-3)
Supplement: Supplementary file 3 — Reporting Summary [file 41467_2023_42232_MOESM3_ESM.pdf]

nature portfolio

Corresponding author(s): Andreas Hartmann
Michael Schlierf

Last updated by author(s): Sep 6, 2023

Reporting Summary

Nature Portfolio wishes to improve the reproducibility of the work that we publish. This form provides structure for consistency and transparency in reporting. For further information on Nature Portfolio policies, see our Editorial Policies and the Editorial Policy Checklist.

Statistics

For all statistical analyses, confirm that the following items are present in the figure legend, table legend, main text, or Methods section.

n/a	Confirmed	
☐	☒	The exact sample size (n) for each experimental group/condition, given as a discrete number and unit of measurement
☐	☒	A statement on whether measurements were taken from distinct samples or whether the same sample was measured repeatedly
☒	☐	The statistical test(s) used AND whether they are one- or two-sided<br>*Only common tests should be described solely by name; describe more complex techniques in the Methods section.*
☒	☐	A description of all covariates tested
☒	☐	A description of any assumptions or corrections, such as tests of normality and adjustment for multiple comparisons
☐	☒	A full description of the statistical parameters including central tendency (e.g. means) or other basic estimates (e.g. regression coefficient) AND variation (e.g. standard deviation) or associated estimates of uncertainty (e.g. confidence intervals)
☒	☐	For null hypothesis testing, the test statistic (e.g. F, t, r) with confidence intervals, effect sizes, degrees of freedom and P value noted<br>*Give P values as exact values whenever suitable.*
☒	☐	For Bayesian analysis, information on the choice of priors and Markov chain Monte Carlo settings
☒	☐	For hierarchical and complex designs, identification of the appropriate level for tests and full reporting of outcomes
☒	☐	Estimates of effect sizes (e.g. Cohen's d, Pearson's r), indicating how they were calculated

Our web collection on statistics for biologists contains articles on many of the points above.

Software and code

Policy information about availability of computer code

Data collection	Raw datasets are available on OpARA: DNA ruler (http://dx.doi.org/10.25532/OPARA-202); DNA hairpin (http://dx.doi.org/10.25532/OPARA-206); S6 protein (http://dx.doi.org/10.25532/OPARA-209); SSB and RecA competition (http://dx.doi.org/10.25532/OPARA-205); CFTR TM3/4 (http://dx.doi.org/10.25532/OPARA-207); Raw data presented in figures are available in the source data file.
Data analysis	For data analysis please use the autoFRET package https://github.com/SchlierfLAB/autoFRET and http://dx.doi.org/10.5281/zenodo.8269134. The associated wiki provides an installation guide and a demonstration for data analysis and visualization

For manuscripts utilizing custom algorithms or software that are central to the research but not yet described in published literature, software must be made available to editors and reviewers. We strongly encourage code deposition in a community repository (e.g. GitHub). See the Nature Portfolio guidelines for submitting code & software for further information.

Data

Policy information about availability of data

All manuscripts must include a data availability statement. This statement should provide the following information, where applicable:

- Accession codes, unique identifiers, or web links for publicly available datasets
- A description of any restrictions on data availability
- For clinical datasets or third party data, please ensure that the statement adheres to our policy

Raw datasets are available on OpARA: DNA ruler (http://dx.doi.org/10.25532/OPARA-202); DNA hairpin (http://dx.doi.org/10.25532/OPARA-206); S6 protein (http://dx.doi.org/10.25532/OPARA-209); SSB and RecA competition (http://dx.doi.org/10.25532/OPARA-205); CFTR TM3/4 (http://dx.doi.org/10.25532/OPARA-207); Raw data presented in figures are available in the source data file.

Human research participants

Policy information about studies involving human research participants and Sex and Gender in Research.

Reporting on sex and gender	n.a.
Population characteristics	n.a.
Recruitment	n.a.
Ethics oversight	n.a.

Note that full information on the approval of the study protocol must also be provided in the manuscript.

Field-specific reporting

Please select the one below that is the best fit for your research. If you are not sure, read the appropriate sections before making your selection.

☒ Life sciences ☐ Behavioural & social sciences ☐ Ecological, evolutionary & environmental sciences

For a reference copy of the document with all sections, see nature.com/documents/nr-reporting-summary-flat.pdf

Life sciences study design

All studies must disclose on these points even when the disclosure is negative.

Sample size	The sample size varied from data set to data set depending on the final concentration of double labeled molecules. A coarse estimate given the labeling efficiency and acquisition time of 20 min per well was done based on previous studies in the lab. Exact experimental conditions are given in the Methods.
Data exclusions	All data were visualized and filtered using the pyVIZ software package (https://github.com/SchlierfLAB/autoFRET). Data exclusion criteria are listed in the Supplementary Information.
Replication	Replication was used to quantify the accuracy and precision of the automation approach as shown in Fig. 2C and extensively discussed. Replication was not used for the other data sets.
Randomization	We did not test a hypothesis, therefore a randomization was not required.
Blinding	We did not test a hypothesis, therefore a blinding was not required.

Reporting for specific materials, systems and methods

We require information from authors about some types of materials, experimental systems and methods used in many studies. Here, indicate whether each material, system or method listed is relevant to your study. If you are not sure if a list item applies to your research, read the appropriate section before selecting a response.

Materials & experimental systems

n/a	Involved in the study	
☒	☐	Antibodies
☒	☐	Eukaryotic cell lines
☒	☐	Palaeontology and archaeology
☒	☐	Animals and other organisms
☒	☐	Clinical data
☒	☐	Dual use research of concern

Methods

n/a	Involved in the study	
☒	☐	ChIP-seq
☒	☐	Flow cytometry
☒	☐	MRI-based neuroimaging
